# Mining the Factors Driving the Evolution of the Pit Mud Microbiome under the Impact of Long-Term Production of Strong-Flavor Baijiu

**DOI:** 10.1128/AEM.00885-21

**Published:** 2021-08-11

**Authors:** Li-Juan Chai, Wei Qian, Xiao-Zhong Zhong, Xiao-Juan Zhang, Zhen-Ming Lu, Su-Yi Zhang, Song-Tao Wang, Cai-Hong Shen, Jin-Song Shi, Zheng-Hong Xu

**Affiliations:** a Key Laboratory of Industrial Biotechnology of Ministry of Education, School of Biotechnology, Jiangnan University, Wuxi, People's Republic of China; b National Engineering Laboratory for Cereal Fermentation Technology, Jiangnan University, Wuxi, People's Republic of China; c School of Pharmaceutical Science, Jiangnan University, Wuxi, People's Republic of China; d Jiangsu Engineering Research Center for Bioactive Products Processing Technology, Jiangnan University, Wuxi, People's Republic of China; e National Engineering Research Center of Solid-State Brewing, Luzhou, People's Republic of China; University of Helsinki

**Keywords:** Chinese strong-flavor baijiu, different-aged pit mud, microbial biomass, prokaryotic communities, volatile metabolites

## Abstract

The mud cellar creates a unique microenvironment for the fermentation of strong-flavor baijiu (SFB). Recent research and long-term practice have highlighted the key roles of microbes inhabiting pit mud in the formation of SFB’s characteristic flavor. A positive correlation between the quality of SFB and cellar age was extracted from practice; however, the evolutionary patterns of pit mud microbiome and driving factors remain unclear. Here, based on the variation regularity analysis of microbial community structure and metabolites of samples from cellars of different ages (∼30/100/300 years), we further investigated the effects of lactate and acetate (main microbial metabolites in fermented grains) on modulating the pit mud microbiome. Esters (50.3% to 64.5%) dominated the volatile compounds identified in pit mud, and contents of the four typical acids (lactate, hexanoate, acetate, and butyrate) increased with cellar age. Bacteria (9.5 to 10.4 log_10_ [lg] copies/g) and archaea (8.3 to 9.1 lg copies/g) mainly constituted pit mud microbiota, respectively dominated by *Clostridia* (39.7% to 81.2%) and *Methanomicrobia* (32.8% to 92.9%). An upward trend with cellar age characterized the relative and absolute abundance of the most predominant bacterial and archaeal genera, *Caproiciproducens* and *Methanosarcina*. Correlation analysis revealed significantly (*P* < 0.05) positive relationships between the two genera and major metabolites. Anaerobic fermentation with acetate and lactate as carbon sources enhanced the enrichment of *Clostridia*, and furthermore, the relative abundance of *Caproiciproducens* (40.9%) significantly increased after 15-day fed-batch fermentation with lactate compared with the initial pit mud (0.22%). This work presents a directional evolutionary pattern of pit mud microbial consortia and provides an alternative way to accelerate the enrichment of functional microbes.

**IMPORTANCE** The solid-state anaerobic fermentation in a mud cellar is the most typical feature of strong-flavor baijiu (SFB). Metabolites produced by microbes inhabiting pit mud are crucial to create the unique flavor of SFB. Accordingly, craftspeople have always highlighted the importance of the pit mud microbiome and concluded by centuries of practice that the production rate of high-quality baijiu increases with cellar age. To deepen the understanding of the pit mud microbiome, we determined the microbial community and metabolites of different-aged pit mud, inferred the main functional groups, and explored the forces driving the microbial community evolution through metagenomic, metabolomic, and multivariate statistical analyses. The results showed that the microbial consortia of pit mud presented a regular and directional evolutionary pattern under the impact of continuous batch-to-batch brewing activities. This work provides insight into the key roles of the pit mud microbiome in SFB production and supports the production optimization of high-quality pit mud.

## INTRODUCTION

Chinese liquor (i.e., baijiu), a kind of traditionally distilled spirits, is produced in a naturally open environment. The conversion of cereals (mainly sorghum) to ethanol and thousands of flavor substances accomplished by multiple microorganisms is the most typical characteristic of baijiu’s spontaneous fermentation process (1, 2). The production of baijiu reached 7.86 million kiloliters in 2019, with a revenue of ∼83.43 billion dollars according to the reports from the China Industrial Information Network (http://www.chyxx.com/), and strong-flavor baijiu makes up >70% of the total production. The solid-state anaerobic fermentation process of strong-flavor baijiu occurs in an underground mud cellar (see Fig. S1 in the supplemental material) and lasts for 2 to 4 months mainly depending on the manufacturing technology and geographic environment (3). The abundant and representative flavor compounds of strong-flavor baijiu include ethyl hexanoate, ethyl lactate, ethyl acetate, and ethyl butyrate and their corresponding organic acid precursors (4). According to the national standard of strong-flavor baijiu (number GB/T 10781.1-2006) (5), the concentrations of total esters and ethyl hexanoate are set as key parameters to classify the quality grade. Both recent research and long-term production practice have highlighted the crucial roles of microbes inhabiting pit mud in the accumulation of typical flavor compounds (mainly hexanoic acid and butyric acid) of strong-flavor baijiu (6–8). Thus, elucidating the features of the pit mud microbial community structure and function will provide key information to understand the complex flavor formation mechanisms of strong-flavor baijiu.

A fermentation mud cellar provides two spatially linked habitats for microbial consortia, including fermented grains (termed *jiupei* in Chinese) and pit mud. *Lactobacillus* dominated the prokaryotic communities of fermented grains during fermentation of strong-flavor baijiu (9), while the predominant genera in pit mud included *Hydrogenispora*, *Sedimentibacter*, *Clostridium*, and *Caproiciproducens*, which are affiliated with the class *Clostridia* (10). The presence of highly abundant lactic acid bacteria led to a large amount of lactic acid accumulated in fermented grains, even higher than ethanol content (11). With the degradation of starch and other macromolecules, *huangshui*, namely, the leaching solution of fermented grains, is accumulated in the fermentation cellar, which contains lots of microbial metabolites, such as lactic acid, ethanol, and acetic acid (12). When *huangshui* comes into contact with pit mud, these compounds can be further metabolized by pit mud microbiota. Culture-independent (e.g., metagenomic and amplicon sequencing analysis) and -dependent approaches revealed that the producers of hexanoic acid and butyric acid mainly existed in pit mud, further scattered in *Clostridia*, such as clostridial cluster IV and *Clostridium* (6, 7, 13). Clostridial microbes can utilize ethanol, lactic acid, and acetic acid as substrates to synthesize hexanoic acid and butyric acid (13–15). A microbial community obtained from pit mud preferred to use lactic acid predominantly for hexanoic acid production rather than ethanol or glucose (16). With the increase of usage time of a fermentation cellar, the abundance of *Lactobacillus* significantly decreased in 50-year pit mud compared with the new samples, and the levels of major genera belonging to *Clostridia* increased (17). So far, the underlying mechanisms that illustrate the temporal changing patterns of pit mud microbial community under the impacts of continuous multibatch fermentation remain unclear.

In addition, the diversity and abundance of *Clostridia* were significantly higher in normal and high-quality pit mud than in the degraded samples, while *Lactobacillus* decreased with the improvement of quality (18). Accordingly, a major goal of pit mud production is to increase the levels of clostridial microbes to meet the target of hexanoic acid accumulation enhancement, which will further help improve product flavor (8, 19). The main objective of this work was to explore the forces modulating the pit mud microbiome on the basis of the microbial community structure and metabolic characteristics analysis of different-aged samples. Microbial communities of pit mud samples from ∼30/100/300-year-old cellars were scanned by quantitative PCR (qPCR), and the prokaryotic communities were further investigated through amplicon sequencing. Metabonomics combined with multivariate statistical analysis was performed to reveal the functional microbial groups. Then, we explored the impacts of lactic acid and acetic acid on the microbial community and main metabolites of pit mud. This research promotes the understanding of factors driving the temporal dynamics of pit mud microbial communities and provides an alternative way for the production of high-quality pit mud.

## RESULTS

### Variation of physicochemical properties of different-aged pit mud.

**FIG 1:**
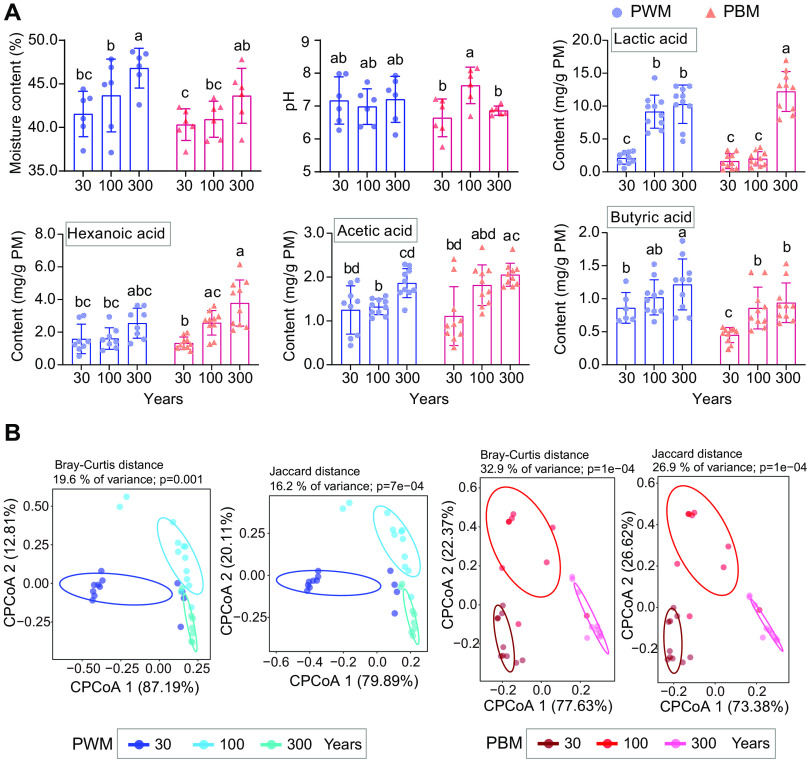
The physicochemical properties of pit wall/bottom mud (PWM/PBM) samples from different-aged fermentation cellars (∼30, ∼100, and ∼300 years). (A) Moisture content, pH, and the contents of the four main organic acids, including lactic acid, hexanoic acid, acetic acid, and butyric acid. (B) Constrained principal coordinate analysis (CPCoA) based on Bray-Curtis and Jaccard distance of the pit mud microbial volatile metabolites.

The moisture content of pit-wall mud (PWM) and pit-bottom mud (PBM) samples from different-aged fermentation cellars (about 30, 100, and 300 years) ranged from ∼40% to ∼46%, and the pH of these samples was around 7.0 (Fig. 1A). Hexanoic acid, lactic acid, acetic acid, and butyric acid are the essential flavoring compounds of Chinese strong-flavor baijiu. Accordingly, the accumulation of these four organic acids in pit mud was determined, which presented an obviously ascending trend with the increase of fermentation cellar age (Fig. 1A). In general, the order of these four organic acid contents in both PWM and PBM was as follows: lactic acid > hexanoic acid > acetic acid > butyric acid. Lactic acid content in the 300-year PWM (10.3 mg/g) and PBM (12.2 mg/g) samples, respectively, showed a 4.9- and 7.6-fold rise compared to that in the 30-year samples. Hexanoic acid content in PWM did not differ statistically among different years; however, 100-year (2.6 mg/g) and 300-year PBM (3.8 mg/g) exhibited significantly higher (*P *< 0.05) hexanoic acid accumulation than that of 30-year samples (1.3 mg/g). Similarly, no significant differences were observed in the contents of acetic acid and butyric acid among different-aged PWM samples. Acetic acid content in 300-year (2.1 mg/g) PBM was almost twice that of 30-year samples (1.1 mg/g). Compared with 30-year PBM (0.45 mg/g), butyric acid content increased significantly (*P *< 0.05) in 100-year (0.86 mg/g) and 300-year samples (0.94 mg/g).

A total of 109 volatile compounds were identified by headspace-solid phase microextraction (HS-SPME)/gas chromatography-mass spectrometry (GC-MS) analysis of the studied pit mud samples, and these metabolites were distributed across six chemical classes: esters (67), alcohols (12), acids (9), ketones (5), aldehydes (4), and others (12) (see Fig. S2 in the supplemental material). Esters dominated the volatile compounds in all pit mud samples, accounting for 50.3% to 64.5% of the total peak area, except 30-year PWM samples (see Fig. S3A in the supplemental material). Hexanoic acid, ethyl ester was the most abundant in all volatiles, constituting 23.7% to 30.7% in the tested samples (10.2% in 30-year PWM) (Fig. S3B). The second abundant ester, hexanoic acid, hexyl ester, showed tendency to ascend in pit mud with the increase of years, respectively reaching 13.7% and 15.8% in 300-year PWM and PBM samples. Among volatile acids, hexanoic acid presented the highest content, representing 64.9% to 85.6% of all of the acid peak area. The third predominant class of volatiles was alcohols with 1-hexanol as the major constituent. Compared with samples from 100- and 300-year-old cellars, 30-year samples contained higher quantities of alcohols, especially 30-year PWM (38.7%) (Fig. S3A). On the whole, constrained principal coordinate analysis (CPCoA) based on Bray-Curtis and Jaccard distance revealed clearly distinct profiles of the microbial volatile metabolites of pit mud samples from different-aged cellars (Fig. 1B).

### The overall features of prokaryotic community structure in pit mud.

**FIG 2:**
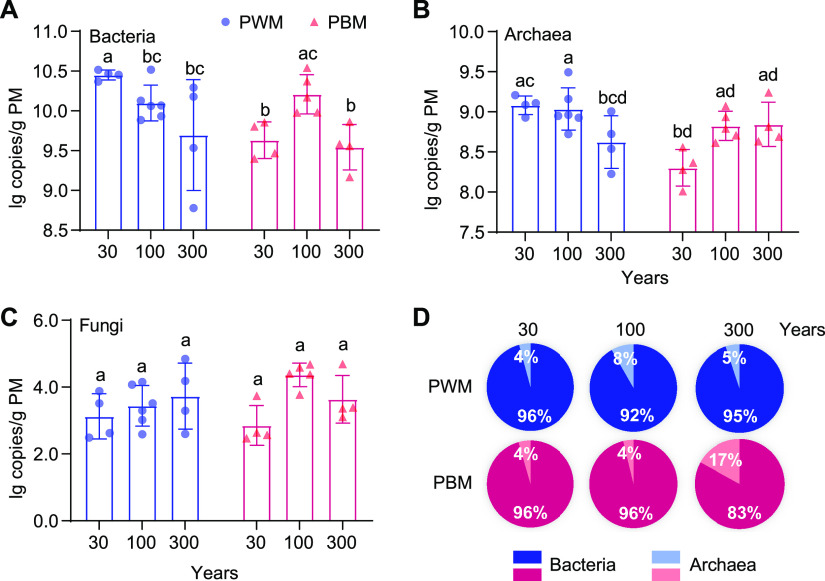
The biomass distribution of bacteria, archaea, and fungi in pit mud by quantitative PCR analysis. Bacterial (A), archaeal (B), and fungal biomass (C) and their proportions (D).

The biomass distribution of bacteria, archaea, and fungi in pit mud was determined via quantitative PCR (qPCR). The copy numbers of the bacterial 16S rRNA gene of PWM samples decreased from 10.4 (30-year) to 9.7 (300-year) log_10_ (lg) copies/g pit mud, yet regarding PBM, 100-year samples (10.2 lg copies/g) were with significantly higher values (*P* < 0.05) than 30-year (9.6 lg copies/g) and 300-year (9.5 lg copies/g) samples (Fig. 2A). Archaeal biomass in PWM samples was on the decline over time, from 9.1 lg copies/g in 30-year samples to 8.6 lg copies/g in 300-year samples (Fig. 2B). Conversely, archaeal biomass appeared to be higher in 100-year (8.8 lg copies/g) and 300-year (8.8 lg copies/g) PBM samples than 30-year (8.3 lg copies/g) samples. The fungal abundance varying from 2.8 to 4.4 lg copies/g exhibited no significant differences across all samples (Fig. 2C). Overall, bacteria and archaea made up almost all of the biomass across all samples regardless of the sampling sites and fermentation cellar ages (Fig. 2D). Thus, we subsequently focused on exploring the temporal dynamic features of prokaryotic communities in pit mud through 16S rRNA gene amplicon sequencing.

Using a cutoff of 97% similarity of 16S rRNA gene sequences (see Table S1 in the supplemental material), 1,563 bacterial operational taxonomic units (OTUs) and 78 archaeal OTUs were clustered. The alpha diversity of both bacterial and archaeal communities in pit mud showed different variation patterns over time between PWM and PBM samples (see Fig. S4 in the supplemental material). Bacterial and archaeal community richness increased with cellar age in PWM samples but was not statistically significant. As for PBM samples, bacterial community richness of 30-year samples was just slightly higher than 100-year samples but significantly higher (*P* < 0.05) than 300-year samples, and archaeal community richness had not shifted noticeably. For both PWM and PBM, bacterial community diversity was the highest in 100-year samples. The highest diversity of the archaeal community was also observed in 100-year PWM samples; however, archaeal diversity decreased markedly with increasing cellar age for PBM samples. Furthermore, principal coordinate analysis (PCoA) at the OTU level depending on Bray-Curtis and Jaccard distance clearly revealed the distinctive prokaryotic community structures among different-aged pit mud samples (see Fig. S5 in the supplemental material).

### Temporal evolution patterns of prokaryotic communities in pit mud.

**FIG 3:**
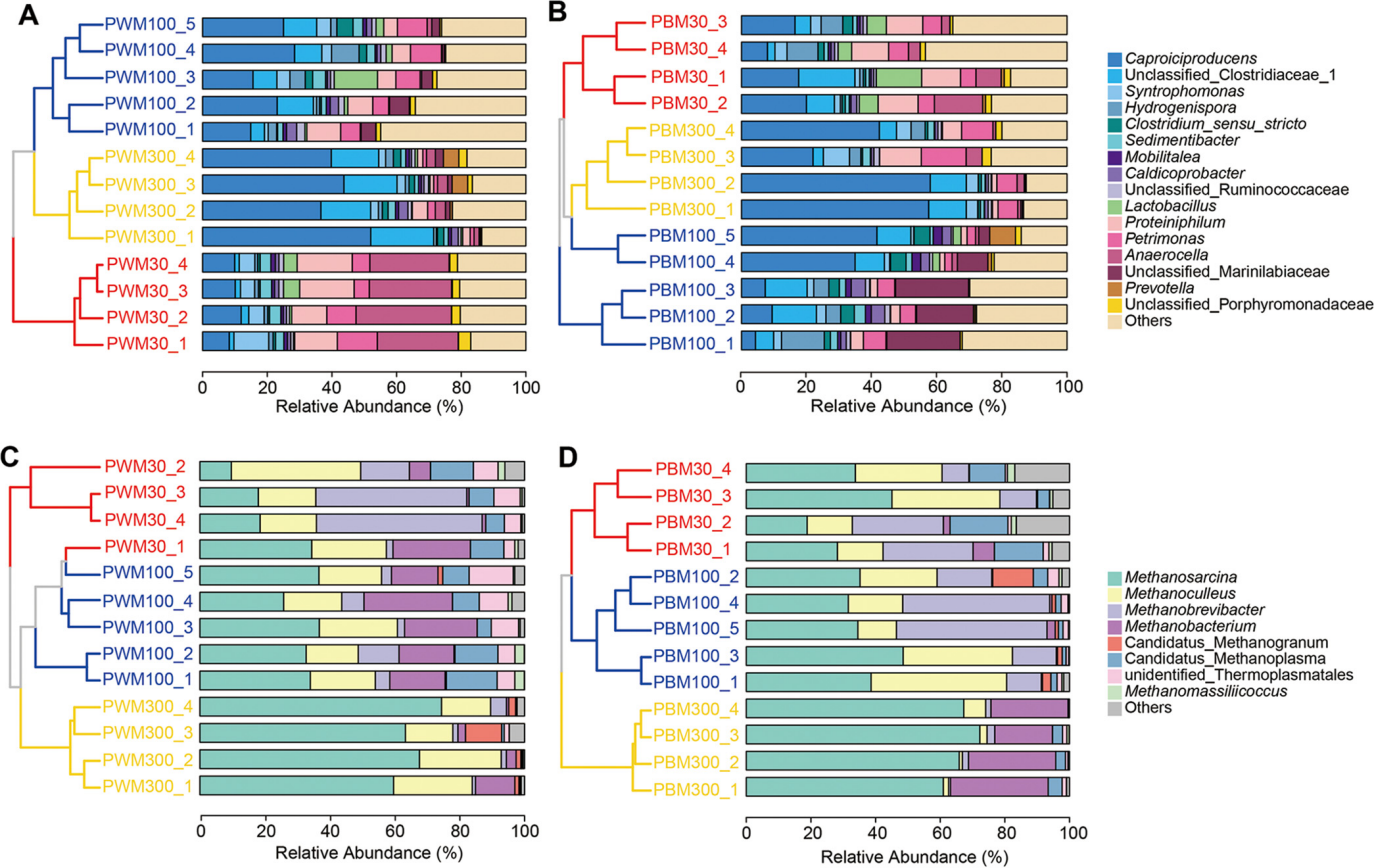
Distribution of the abundant genera (average relative abundance > 1%) of different-aged pit mud prokaryotic communities. The relative abundances of bacterial/archaeal genera in pit wall mud (PWM) (A and C) and pit bottom mud (PBM) (B and D) samples. Numbers 30, 100, and 300 represent the age of fermentation cellar; numbers 1 through 5 represent the biological replicates.

The bacterial consortia in pit mud were dominated by the phyla *Firmicutes* and *Bacteroidetes*, respectively representing 40.9% to 91.0% and 7.1% to 55.3% of total abundance in each sample (see Fig. S6A and B in the supplemental material). At the class level, bacteria were mainly found within *Clostridia* and *Bacteroidia*. The relative abundance of *Clostridia* increased with cellar age in both PWM and PBM samples—average relative abundance (ARA) of 30-, 100-, and 300-year PWM (PBM) was 39.7% (46.0%), 65.5% (65.1%), and 81.2% (73.4%), respectively. Meanwhile, the average relative abundance of *Bacteroidia* decreased from 51.9% (26.8%) in 30-year PWM (PBM) to 14.4% (18.3%) in their corresponding 300-year samples. Regarding the genus level, bacteria inhabiting pit mud were mainly populated by 16 groups (ARA > 1%), which comprised 55.1% to 87.5% of the bacterial community in each sample (Fig. 3A and B). *Caproiciproducens* was the most abundant genus across all samples, except 30-year PWM dominated by *Anaerocella* with ARA of 26.1%. In the 30-year PWM (PBM) samples, the ARA of *Caproiciproducens* was 10.0% (15.5%), which rose to 21.4% (19.5%) in 100-year samples and further reached 43.0% (45.0%) in 300-year samples. Other predominant genera also showed diverse variation patterns with the increasing cellar age. The ARA of unclassified *Clostridiaceae* 1 in PWM rocketed from 1.7% in 30-year samples to 8.3% in 100-year and subsequently reached 16.5% in 300-year samples, while its ARA remained relatively stable in different-aged PBM samples (7.8% to 10.3%). *Syntrophomonas* constituted 6.2% in 30-year PWM and dropped to 2.7% and 2.0% in 100- and 300-year samples; nevertheless, for PBM, the highest ARA was found in 300-year samples (4.9%). The ARAs of *Hydrogenispora* and *Clostridium sensu stricto* in 100-year samples were the highest no matter for PWM or PBM. Another noteworthy phenomenon was that the ARA of *Lactobacillus* visibly decreased while cellar age increased, particularly for PBM samples, which was downregulated from 7.5% in 30-year samples to 0.23% in 300-year samples.

The phylum *Euryarchaeota* made up 99.9% of the pit mud archaeal community in most of the studied samples, mainly scattered in classes *Methanomicrobia* (32.8% to 92.9%), *Methanobacteria* (4.6% to 52.2%), and *Thermoplasmata* (2.6% to 24.4%) (Fig. S6C and D). According to the ARA per sample, the top abundant genera included *Methanosarcina* (42.0%), *Methanoculleus* (19.3%), *Methanobrevibacter* (14.2%), and *Methanobacterium* (10.0%). In both PWM and PBM, an upward trend characterized the relative abundance of *Methanosarcina* with the increasing of cellar age—the ARA of 30-, 100-, and 300-year PWM (PBM) was 20.2% (31.5%), 33.2% (37.7%), and 66.3% (66.6%), respectively (Fig. 3C and D). *Methanoculleus* maintained the relative abundance in different-aged PWM samples at around 20%, but for PBM, it had fallen by nearly 9 fold in 300-year samples (ARA, 2.8%) compared with that of 30- (22.0%) and 100-year samples (25.7%). The relative abundance of *Methanobrevibacter* showed a variation pattern similar to that of *Methanoculleus* in PBM. The highest relative abundance of *Methanobacterium* appeared in 300-year PBM (24.7%), which was significantly higher than the corresponding 30- (2.3%) and 100-year samples (0.84%), but for PWM, 100-year samples (19.6%) owned the maximum ARA.

The absolute abundance of a species can be quantified by the multiplication of relative abundance and total bacterial 16S rRNA gene copies when its relative abundance was >10% (20). Here, we calculated the absolute concentrations of the most abundant bacterial and archaeal genera, *Caproiciproducens* and *Methanosarcina* (see Fig. S7 in the supplemental material). Their absolute concentrations didn’t show notable changes in different-aged PWM samples. For PBM, the absolute abundance of *Caproiciproducens* was the highest in 100-year samples (9.3 lg copies/g), which was, respectively, 6.3 and 3.1 times that when compared with 30- (8.8 lg copies/g) and 300-year samples (9.1 lg copies/g), and the absolute abundance of *Methanosarcina* exhibited an upward trend with the increase of cellar age, which was, respectively, 7.8, 8.4, and 8.7 lg copies/g in 30-, 100-, and 300-year samples and consistent with the changing trend of relative abundance.

Moreover, unweighted pair group method with arithmetic mean (UPGMA) average linkage clustering analysis revealed that prokaryotic communities of different-aged pit mud had a clear time scale hierarchical structure (Fig. 3), demonstrating the distinctive composition and diversity of pit mud microbiota among different-aged cellars together with the above-mentioned PCoA analysis (Fig. S5). Linear discriminant analysis effect size (LEfSe) analysis revealed that the potential biomarkers at the genus level for 30-year pit mud samples included *Syntrophomonas*, *Sedimentibacter*, *Lactobacillus*, *Proteiniphilum*, *Petrimonas*, and *Anaerocella*; for 100-year samples, they were scattered in *Methanoculleus*, *Methanobrevibacter*, *Clostridium sensu stricto*, and *Caldicoprobacter*; for 300-year samples, members affiliated with *Methanosarcina*, *Methanobacterium*, *Caproiciproducens*, unclassified *Clostridiaceae* 1, and *Prevotella* were more enriched and discriminatory (see Fig. S8 in the supplemental material).

### Relationships between abundant prokaryotic taxa and major microbial metabolites.

**FIG 4:**
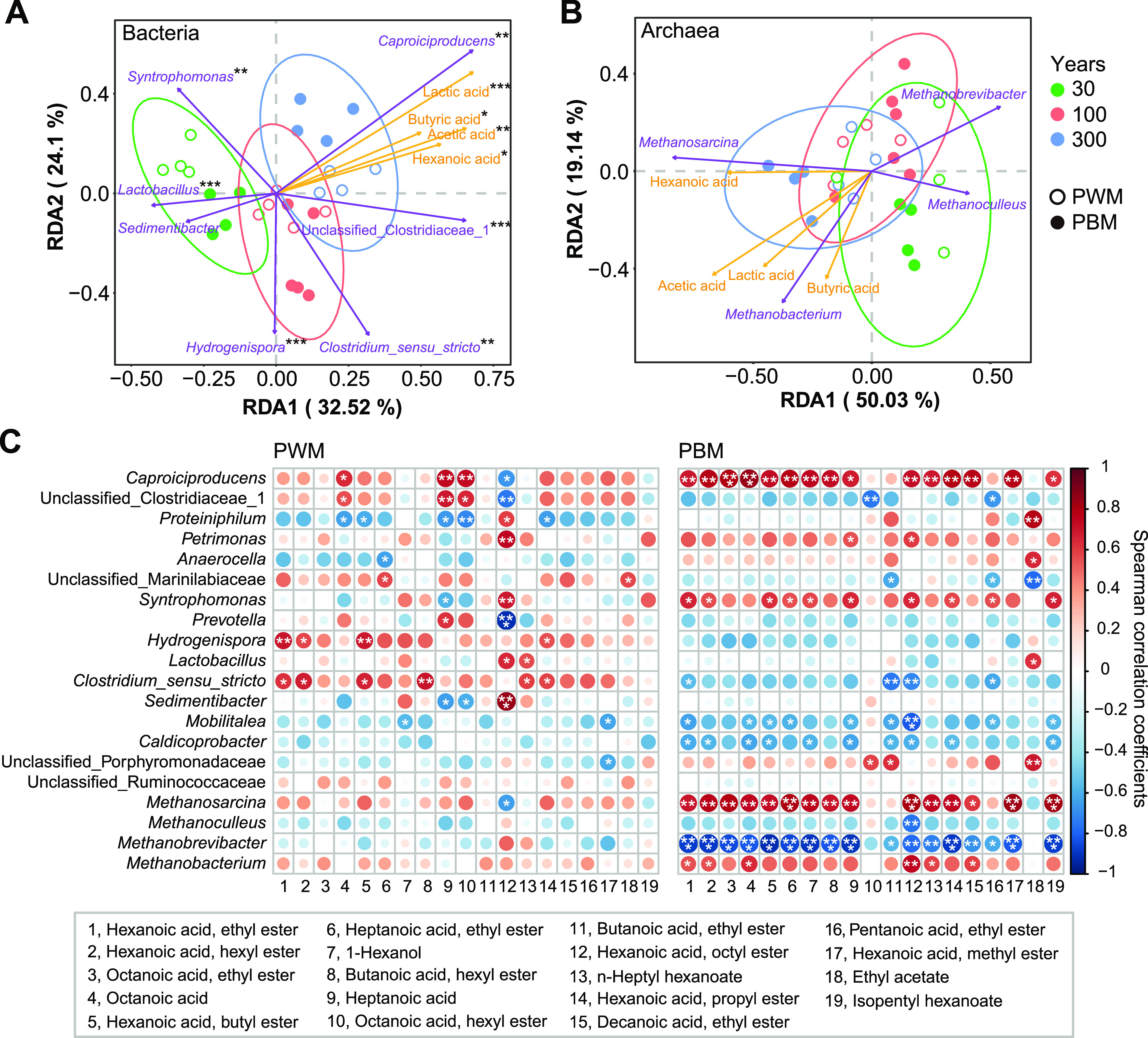
Correlation analysis between predominant prokaryotic genera (average relative abundance > 1%) and main metabolites. Redundancy analysis (RDA) for the relationship between bacterial (A) or archaeal (B) genera (average relative abundance > 2%) and the four main organic acids including lactic acid, hexanoic acid, acetic acid, and butyric acid. *, adjusted *P* value < 0.05; **, adjusted *P* < 0.01; ***, adjusted *P* < 0.001. (C) Spearman correlation coefficients between predominant prokaryotic genera and major volatile compounds (average relative concentration > 50 mg/kg). *, *P* value < 0.05; **, *P* < 0.01; ***, *P* < 0.001.

Redundancy analysis (RDA) was employed to depict the correlations between the four major organic acids and predominant microbial taxa. As shown in the RDA ordination plot, axes 1 and 2, respectively, explained 32.5% and 24.1% of the total bacterial variance (Fig. 4A). The bacterial communities in different-aged pit mud samples were clearly separated b*y* axis 1. The significant factors (adjusted *P *< 0.05) contributing to the variance of 30- (*Syntrophomonas* and *Lactobacillus*), 100- (*Clostridium sensu stricto* and *Hydrogenispora*), and 300-year (*Caproiciproducens* and unclassified *Clostridiaceae* 1) pit mud bacterial communities were distinctive, which were also identified as the potential biomarkers for each group by LEfSe analysis (Fig. S8). Moreover, *Caproiciproducens* and unclassified *Clostridiaceae* 1 were positively correlated with the four major organic acids (Fig. 4A). The first two axes explained 69.1% of the total variation in the archaeal community (Fig. 4B). When using an adjusted *P *value of <0.05 as a cutoff, no significant contributors were found; however, according to the *P* value, lactic acid (*P = *0.003), hexanoic acid (0.039), acetic acid (0.048), and *Methanosarcina* (0.018) significantly influenced the total archaeal variance. Positive correlations were found between *Methanosarcina* and *Methanobacterium* and the four major organic acids, in particular, *Methanosarcina* and hexanoic acid. Furthermore, we calculated the Spearman correlation coefficients between the predominant microbial taxa and volatile metabolites (Fig. 4C). *Caproiciproducens* and *Methanosarcina* exhibited significantly positive correlations with almost all of the tested volatiles in PBM, while *Methanobrevibacter* was negatively correlated to them. As for PWM, *Clostridium sensu stricto* and *Hydrogenispora* were positively related with the volatiles. Generally, more significant correlations between microbes and volatile compounds were observed in PBM samples than in PWM samples, inferring that the PBM microbial consortia may play more important roles in the flavor formation of strong-aroma baijiu (Fig. 4C).

### Distinct bacterial community structures of pit mud following anaerobic fermentation with acetate and lactate.

**FIG 5:**
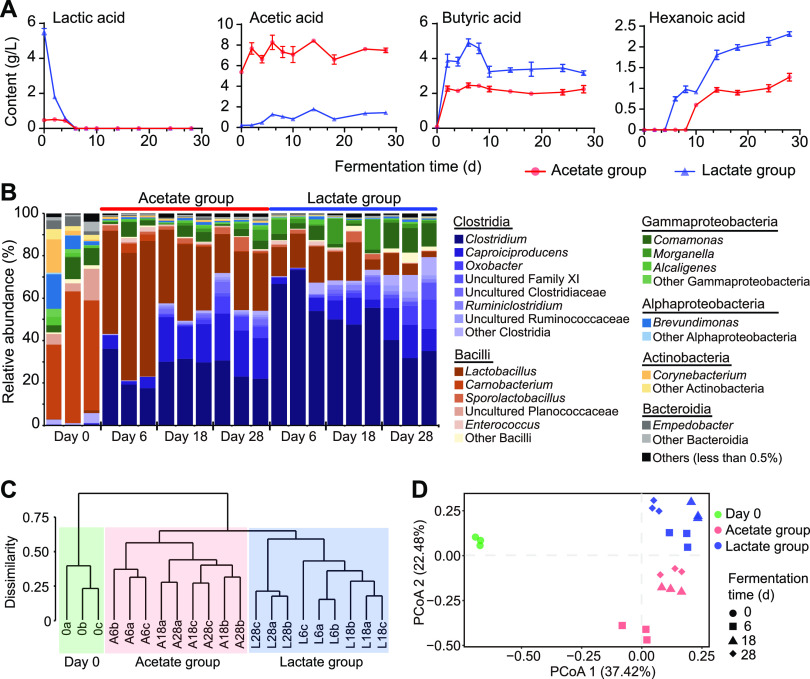
The effects of acetate and lactate on pit mud bacterial community and main organic acids. (A) Changes of lactic acid, acetic acid, butyric acid, and hexanoic acid contents in the fermentation system. (B) Temporal dynamics of bacterial community structure of pit mud during fermentation. Cluster analysis (C) and principal coordinate analysis (PCoA) (D) of the pit mud bacterial community based on Bray-Curtis algorithms.

The influences of different carbon resources, including acetate and lactate, on the structure and performance of pit mud bacterial community were investigated. Glucose in the fermentation broth was quickly consumed up within 1 day. The determination of the main organic acids in the two studied groups, respectively, using acetate and lactate as the carbon sources was conducted by high-performance liquid chromatography (HPLC) (Fig. 5A). Lactate was used up on day 6 for both of the two groups, and acetate content stayed moderately altered during the anaerobic fermentation, which was 7.5 g/liter in the acetate group and 1.4 g/liter in the lactate group on day 28. Using lactate as the carbon source was more conducive to the accumulation of butyrate and hexanoate. The content of butyrate reached 2.3 g/liter on day 2 in the acetate group and kept stable until the end, while for the lactate group, butyrate content peaked on day 6 (4.9 g/liter) and then decreased and remained at 3.2 to 3.6 g/liter after day 10. Hexanoate could be detected from day 6 and 10 in the lactate and acetate groups, respectively, presenting an upward trend during fermentation and accumulating to 2.3 g/liter in the lactate group and 1.3 g/liter in the acetate group on day 28.

In general, bacterial biomass was ∼75-fold more than archaeal biomass for the two groups, and fungi were not identified in the fermentation broth probably due to the anaerobic conditions (see Fig. S9 in the supplemental material). The 16S rRNA gene copies of bacteria and archaea increased to the maximum on day 6 and then decreased. Bacterial and archaeal biomass was slightly higher on day 28 in the lactate group (bacteria, 8.8 lg copies/g; archaea, 7.3 lg copies/g) than the acetate group (bacteria, 7.9 lg copies/g; archaea, 7.1 lg copies/g). Amplicon sequencing analysis showed that bacterial community richness and diversity were upregulated following the anaerobic fermentation of acetate and lactate compared with that of the initial pit mud (see Table S2 in the supplemental material). The bacterial community of the initial pit mud (i.e., samples on day 0) was dominated by classes *Bacilli* (59.6%), *Gammaproteobacteria* (11.6%), *Actinobacteria* (9.3%), and *Alphaproteobacteria* (8.5%), and the most abundant genus was *Carnobacterium* (49.7%), followed by *Brevundimonas* (8.2%), *Comamonas* (7.2%), and *Corynebacterium* (6.2%) (Fig. 5B). After fermentation with acetate and lactate as carbon sources, *Clostridia* dominated the pit mud bacterial community. As for the acetate group, the ARA of *Lactobacillus*, *Clostridium*, and *Caproiciproducens*, respectively, rocketed from 0.58%, 0.28%, and 0.22% on day 0 to 57.6%, 24.3%, and 4.5% on day 6. During the late stage of the fermentation (day 18 to 28), *Clostridium* maintained an ARA at 22.0% to 31.5%; *Lactobacillus*’s ARA decreased to 24.4%, while that of *Caproiciproducens* increased to 21.0% on day 28. With regard to the lactate group, *Clostridia* dominated the whole process, with relative abundance above 70.0% (Fig. 5B). The ARA of *Clostridium* and *Lactobacillus*, respectively, dropped from 64.7% and 15.6% on day 6 to 35.7% and 6.0% on day 28; however, *Caproiciproducens* rose from 3.4% on day 6 to 13.9% on day 28. Furthermore, cluster analysis and PCoA displayed the clearly distinctive effects of acetate and lactate on the bacterial community structures of pit mud (Fig. 5C and D). Spearman correlation analysis showed that the relative abundance of *Clostridium* (*r *= 0.82; *P *< 0.05) presented a notable positive correlation with butyrate content (see Table S3 in the supplemental material). Hexanoate was significantly (*P *< 0.05) and positively correlated with *Caproiciproducens* (*r *= 0.61), *Oxobacter* (*r *= 0.87), *Ruminiclostridium* (*r *= 0.92), and other uncultured bacteria belonging to *Clostridia*, which correlated negatively to lactate except *Caproiciproducens*. There was a significantly positive correlation between *Lactobacillus* and acetate (*r *= 0.72; *P *< 0.05).

### Fed-batch fermentation with lactate as carbon source promoted enrichment of the genus *Caproiciproducens*.

**FIG 6:**
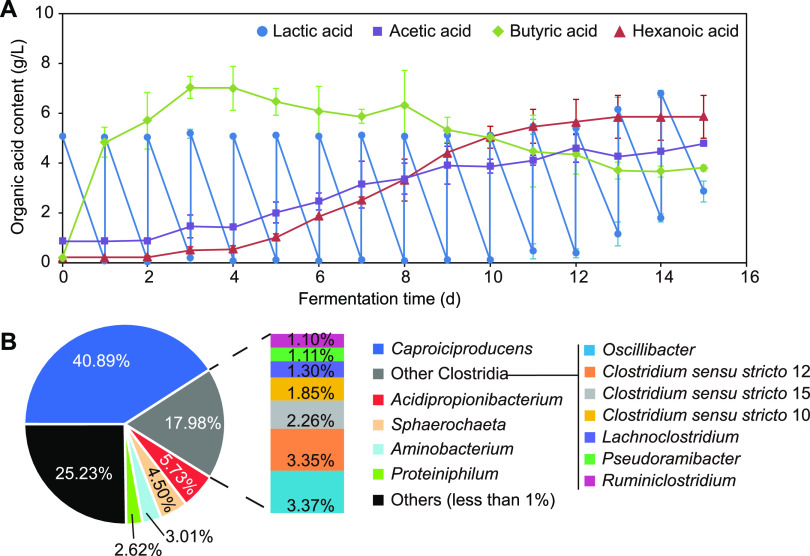
The effects of continuous fermentation with lactate on pit mud bacterial community and main organic acids. (A) Changes of lactic acid, acetic acid, butyric acid, and hexanoic acid contents in the reactor. (B) The composition of pit mud bacterial community after 15 days of fermentation.

The roles of lactate in the evolution of pit mud microbiota were further explored by fed-batch fermentation with lactate as the carbon source due to its better performance in the accumulation of hexanoate and *Clostridia* compared with that of acetate. Lactate could be completely utilized within 1 day in the first 10 days, and later, it could not be used up in 1 day, especially after day 12 (Fig. 6A). Butyrate accumulated rapidly in the early stage of fermentation, reached the maximum on day 3 (7.0 g/liter), decreased subsequently, and remained stable after day 13 (∼3.7 g/liter). The content of hexanoate began to increase on day 3 (0.51 g/liter), and the fasted accumulation rate occurred from day 4 to day 9 (1.5 g/liter/day), reaching the highest value on day 13 (5.9 g/liter). Acetate content showed a moderately and continuously increasing trend during fermentation and was higher than hexanoate content at early stage (days 0 to 8) but lower at late stage (days 9 to 15).

The composition of the pit mud bacterial community was analyzed after 15 days of fermentation (Table S2). A total of 112 genera were identified, and the most predominant genus was *Caproiciproducens* (40.9%), followed by *Clostridium sensu stricto* (7.5%), *Acidipropionibacterium* (5.7%), and *Sphaerochaeta* (4.5%) (Fig. 6B). A 185-fold increase in the relative abundance of *Caproiciproducens* was observed compared with that of the initial pit mud (0.22%).

## DISCUSSION

The production of Chinese baijiu undergoes a solid-state or semi-solid-state fermentation process under an open environment, converting starchy raw materials (mainly sorghum and wheat) to ethanol and diverse flavor compounds through the synergism of multiple microorganisms. Due to the distinctiveness of fermentation containers (e.g., earthen jars, mud pits, and stony pits), brewing techniques, etc., different flavor types of baijiu present their own unique flavor profiles (3, 4). As for strong-flavor baijiu, fermentation carried out in a mud cellar is the most typical feature, and the metabolites produced by microbes colonizing the pit mud are the key compounds to create its unique flavor (6, 7). Therefore, baijiu-making craftspeople have always highlighted the importance of the pit mud microbiome and conclude from the practice that the production rate of high-quality baijiu increases with cellar age. Accordingly, researchers developed some techniques to modulate the pit mud microbiome to promote the flavor and quality of strong-flavor baijiu (8, 19). Pit mud microbial community evolution is driven by the continuous batch-to-batch brewing activities, and with the development of culture and molecular biology techniques, the evolutionary patterns of pit mud microbiota are gradually unveiling (17, 21). Here, we studied the divergent profiles of different-aged pit mud microbiota and microbial major metabolites, inferred the main functional microbial populations, and explored the selective pressures for pit mud microbial community evolution.

As an anaerobic and nearly neutral pH environment, at the class level, the bacterial community of pit mud was dominated by *Clostridia* belonging to the phylum *Firmicutes* regardless of fermentation cellar age (including several years, decades, or even hundreds of years) and geographical location (including Sichuan, Anhui, and Jiangsu Province), followed by *Bacteroidia* in the phylum *Bacteroidetes* (17, 21–23). However, the distribution and composition of these bacterial groups in pit mud were notably divergent with the age of the fermentation cellar. According to our results, the relative abundance of *Clostridia* in pit mud increased while *Bacteroidia* decreased with the increasing of cellar age, which was consistent with the previous report (21). The abundances of *Clostridia* and *Bacteroidia* could be used as potential indicators to distinguish the quality of pit mud (18). The most predominant genus, *Caproiciproducens*, was classified as a novel genus within *Clostridium* cluster IV in 2015, and the type species was obtained from an anaerobic digestion reactor (24). *Caproiciproducens* was first reported as a major bacterial group in pit mud (the mixture of PWM and PBM samples) by Liu et al., and they found its ARA in 400-year pit mud increased by 51.4% compared with that in 40-year samples (21). We further found that the ARA of *Caproiciproducens* increased with cellar age in both PWM and PBM (Fig. 3), while the average absolute abundance was relatively stable in different-aged PWM samples, and it was respectively upregulated by about 500% and 100% in 100- and 300-year PBM relative to 30-year samples (see Fig. S7A in the supplemental material). We hypothesized that the distinctiveness of bacterial communities between PWM and PBM was possibly due to the divergent nutrition and environmental conditions. The leaching solution of fermented grains (i.e., *huangshui*) accumulate at the bottom of the cellar under gravity during fermentation, and *huangshui* contains a large amount of fermented grain microbial metabolites (12). Moreover, recent research revealed the notably different prokaryotic communities of PBM samples from various depths—for >50-year PBM, *Caproiciproducens*, *Syntrophomonas*, and *Hydrogenispora* were mainly distributed in the depth of 0 to 1 cm, 1 to 3 cm, and 5 to 7 cm, respectively, and for 6-year PBM, the characteristic groups in 0 to 1 cm and 1 to 5 cm were, respectively, *Lactobacillus* and *Caproiciproducens* (23). *Caproiciproducens* was also identified as the most prevalent bacteria in 20-year pit mud sampled from the producer in Suqian, Jiangsu Province in Mid-Eastern China, followed by *Fermentimonas*, *Fastidiosipila*, and *Ruminiclostridium* (22), which were not identified as the predominant genera in this study. This phenomenon indicates that bacteria in pit mud exhibit strong biogeographic patterns, which is intriguing and valuable to study more to illuminate the impacts of the ecological environment on the microecology of baijiu fermentation.

Hexanoic acid and its derivate ethyl hexanoate are the key flavor substances of strong-flavor baijiu. Microorganisms residing in the pit mud were the main hexanoic acid producers throughout fermentation (10, 25). Metagenomic analysis of a 30-year pit mud microbiome revealed that the potential hexanoic acid-producing microbes were mainly scattered in clostridial cluster IV (i.e., *Ruminococcaceae*, basonym of *Oscillospiraceae*) and *Clostridium* (6, 26). *Caproiciproducens* is affiliated with the family *Oscillospiraceae*, and the most abundant metabolic end products of its type species Caproiciproducens galactitolivorans were hexanoic acid followed by acetic acid and butyric acid (24). The increasing abundance of *Caproiciproducens* occurred with cellar age coupling with the enhanced accumulation of hexanoic acid (Fig. 1A) (17, 21), and *Caproiciproducens* also significantly and positively correlated with the major volatile metabolites (Fig. 4C), indicating that this microbial population might be the important contributors involved in the production of hexanoic acid and other aroma compounds. However, so far pure strains belonging to *Caproiciproducens* have not been isolated from pit mud based on previous literature, blocking the comprehensive illumination of its roles in the flavor formation of baijiu. Conversely, a large number of strains affiliated with *Clostridium* have been isolated from pit mud and fermented grains due to their production capacity of short-chain fatty acids, especially hexanoic acid (7, 13, 27, 28), and most of these strains further fall into *Clostridium* cluster I (also referred to as *Clostridium sensu stricto*) based on the Ribosomal Database Project (29, 30). *Clostridium sensu stricto* was one of the representative groups and showed the highest relative abundance in 100-year pit mud. Clone library analysis based on the genes encoding key enzymes in butyric acid synthesis and microbial pure culture assays demonstrated that *Clostridium sensu stricto* was the main potential butyric acid producer during brewing (7, 27). Butyric acid and its derivates, such as ethyl butyrate, were also the important aroma contributors of strong-flavor baijiu (4). In addition, the relative abundance of *Clostridium* was dramatically higher in high-quality pit mud than the degraded samples (18). Besides *Clostridium sensu stricto*, *Hydrogenispora* was another significant factor contributing to the variance of 100-year pit mud bacterial community (Fig. 4A). Bioaugmentation with Hydrogenispora ethanolica LX-B significantly enhanced the abundance of *Clostridium* and promoted butyric acid and hydrogen accumulation (31). Cooccurrence of *Clostridium sensu stricto* and *Hydrogenispora* with high abundance in aged pit mud could be conducive to explain the increasing accumulation of short-chain fatty acids with cellar age. These findings implied that the interactions among pit mud microorganisms should be introduced when understanding the forces governing the evolution of pit mud microbiota.

Archaea are the second-largest microbial group in pit mud and are dominated by methanogens. The near-neutral pH environment of pit mud can meet the requirements of methanogens for growth conditions (32). As methanogenic archaea are strictly anaerobic, slow-growing, and critical for the culture conditions, at present, merely three strains assigned to Methanobacterium formicicum and Methanobacterium bryantii were isolated from pit mud (33, 34). High-throughput sequencing analysis demonstrated that the predominant methanogens included *Methanosarcina* and *Methanoculleus* affiliated with the class *Methanomicrobia* and *Methanobacterium* and *Methanobrevibacter* affiliated with the class *Methanobacteria* (17, 18), which was consistent with the results of this work. These identified methanogens can utilize H_2_ and CO_2_, and *Methanosarcina* can also use acetic acid and methanol as substrates for methane production. Several genes encoding the enzymes directly involved in both acetoclastic and hydrogenotrophic methanogenesis pathways were identified in pit mud through metagenomic analysis (6). Using fluorescence *in situ* hybridization (FISH), live methanogens belonging to *Methanobacteriales* and *Methanomicrobiales* were detected (35). Proteomic analysis revealed that functional proteins related to methanogenesis were highly expressed in the 300-year pit mud compared with those in 30-year samples (25). The above-mentioned research showed that both acetoclastic and hydrogenotrophic methanogens were present and functional in pit mud. In this study, *Methanosarcina* abundance was dramatically increased with cellar age, and particularly for PBM samples, it was positively correlated with hexanoic acid and main volatiles, indicating the important functions and roles of *Methanosarcina* in the pit mud microbial metabolism. When cocultured with Clostridium kluyveri H068, methanogen 166 could utilize the hydrogen produced by *C. kluyveri* and eliminate the H_2_-mediated feedback inhibition and, thus, facilitated the hexanoic acid production (36). The increasing abundance of acid/hydrogen-producing microbes, such as *Caproiciproducens* and *Clostridium sensu stricto*, may lead to the accumulation of hydrogen in aged pit mud (13, 24), and meanwhile, the increase of methanogens biomass could be propitious to hydrogen consumption. Thereby, higher contents of short-chain fatty acids were detected in aged pit mud than aging samples. Research on the interactions between methanogens and acid-producing microbes should assist in elucidating the complex microecosystem in pit mud.

In baijiu production, how to enrich the acid-producing microbes and accumulate hexanoic acid in pit mud is one of the key issues that researchers have been exploring. Both our results and those of the previous studies revealed that there were more bacteria with hexanoic acid-producing capacity and higher content of hexanoic acid in older pit mud (17, 21). As for new fermentation cellars, the relative abundance of acid-producing microbes, such as *Caproiciproducens* and *Clostridium*, and the contents of short-chain fatty acids were upregulated after continuous batch-to-batch brewing activities (8). So far, the forces driving the microbial community evolution in pit mud with cellar age remains unclear. According to the hexanoic acid metabolic pathways, the synthesis of hexanoic acid is via a carboxylic acid chain elongation process using acetic acid, lactic acid, butyric acid, and/or ethanol as substrates (14). The microbial metabolites in fermented grains mainly included glucose, ethanol, lactic acid, and acetic acid, and these compounds could be utilized by pit mud microbiota as carbon sources. Previous work demonstrated that microbes inhabiting pit mud preferred to metabolize lactic acid rather than ethanol to produce hexanoic acid (16). Thus, we first considered the impacts of lactic acid and acetic acid on the microbial community of pit mud. The enhancement of acid-producing microbes’ relative abundance was observed in pit mud after fermentation with acetate and lactate, and the lactate group showed better performance (Fig. 5). Furthermore, fed-batch fermentation with lactate was conducted to investigate its roles in the pit mud microbiota. Members belonging to *Caproiciproducens* and *Clostridium sensu stricto* were mainly enriched, which were the predominant bacteria and representative groups in old pit mud (Fig. 3 and 4) (17, 21). The most abundant metabolite of *Caproiciproducens* was hexanoic acid (24), and *Clostridium sensu stricto* was the main butyric acid-producing bacteria in baijiu production (7, 27). The above-mentioned results indicated that lactate had a largely positive role in regulating the microbial community of pit mud, providing useful information for the production of artificial high-quality pit mud.

In summary, under the impact of continuous batch-to-batch liquor-making activities for decades or even hundreds of years, the microbial consortia of pit mud presented a regular and directional variation. Microbial groups conducive to the typical flavor formation of strong-flavor baijiu were enriched with the increase of cellar age, in particular, *Caproiciproducens* and *Methanosarcina*. Furthermore, lactic acid, the main microbial metabolite of fermented grains, promoted the enrichment of beneficial functional bacteria in pit mud. This work preliminarily revealed the influential factors associated with the directional evolution of pit mud microbial consortia, which will support the production technique optimization of high-quality pit mud.

## MATERIALS AND METHODS

### Pit mud sampling and analysis of physicochemical properties.

Pit mud samples were collected from a famous strong-flavor baijiu manufacturer located in Luzhou city, Sichuan Province, China (105°29′50″ E, 28°53′47″ N). Four or five fermentation pits that have been in continual use for ∼30, ∼100, and ∼300 years were selected, respectively. As shown in Fig. S1 in the supplemental material, mud samples of pit bottom were collected from the center and four quarter sites by a cylinder-shaped self-made sampler (diameter × height = 2.0  cm × 5 cm), and for the pit wall, the center and two quarter sites of four walls were sampling sites. Samples of pit bottom and wall from each pit were respectively mixed and transferred into a sterile plastic bag as a biological replicate. The samples were frozen and pulverized in liquid nitrogen and stored at −80°C for DNA extraction. For the determination of microbial metabolites, the samples were stored at −20°C.

Moisture content was measured immediately after sampling using a gravimetric method after drying in the oven at 105°C for 4 h. For each sample, 2 g of pit mud was suspended in 20 ml sterile distilled water and agitated at 100 rpm for 3 h at room temperature. The supernatant extract was collected for further analysis by centrifugation at 10,000 × *g* for 15 min. pH was measured using a Mettler Toledo FiveEasy Plus pH/mV meter equipped with an LE438 solid electrode (Mettler Toledo Instruments, Shanghai, China). Organic acids, including lactic acid, acetic acid, butyric acid, and hexanoic acid, and volatile compounds were respectively determined by high-performance liquid chromatography (HPLC) (27) and headspace-solid phase microextraction (HS-SPME)/gas chromatography-mass spectrometry (GC-MS) (37) as described previously. All experiments were performed in triplicate.

### Biomass analysis via quantitative PCR.

DNA of pit mud was extracted using a PowerSoil DNA isolation kit (MOBIO Laboratories, Inc., Carlsbad, CA, USA) according to the manufacturer’s protocol. Qualified DNA was used for the quantitative analysis of microbial biomass in pit mud using the SYBR select master mix (Applied Biosystems, USA) through a CFX Connect real-time PCR detection system (Bio-Rad, USA). The system and procedure of qPCR were detailed in our previous work (38). Primer sets Eub338 (5′-ACTCCTACGGGAGGCAGCAG-3′)/Eub518 (5′-ATTACCGCGGCTGCTGG-3′), ITS1f (5′-TCCGTAGGTGAACCTGCGG-3′)/5.8s (5′-CGCTGCGTTCTTCATCG-3′), and 931F (5′-AAGAATTGGCGGGGGAGCA-3′)/m1100R (5′-BGGGTCTCGCGTCGTTRC-3′) were selected for bacterial, fungal, and archaeal biomass determination, respectively (39, 40), which was analyzed based on the reported algorithm (41). All reactions were performed in triplicate.

### Prokaryotic community analysis of pit mud via 16S rRNA gene amplicon sequencing.

To target the bacterial community structure in pit mud, the universal primer pair 338F (5′-ACTCCTACGGGAGGCAGCA-3′)/806R (5′-GGACTACHVGGGTWTCTAAT-3′) was used to amplify the V3-V4 hypervariable region of the 16S rRNA gene. Primers Arch349F (5′-GYGCASCAGKCGMGAAW-3′) and Arch806R (5′-GGACTACVSGGGTATCTAAT-3′) (V3-V4 region) (42) were used to determine the archaeal community structure. PCR product purification and library construction were performed as described previously (7). After quality control, paired-end (2 × 300 bp) high-throughput sequencing was carried out using the Illumina MiSeq platform.

The obtained sequencing data was processed using the QIIME pipeline (v1.9.1) (43). Raw sequences were first demultiplexed according to their barcodes, and then the barcode and primer sequences were trimmed for the following assembly of forward and reverse reads (i.e., raw tags) by FLASH (v1.2.7) with a minimum 50-bp overlap (44). Dirty data was filtered from raw tags based on Bokulich et al. (45), and removal of chimeric sequences was performed via the UCHIME algorithm (46) to obtain effective tags for downstream analysis. Subsequently, the qualified effective tags from all samples were clustered into operational taxonomic units (OTUs) with 97% identity by USEARCH (v10) (46). The representative sequences of each OTU were selected for taxonomic assignment through alignment against the SILVA and Ribosomal Database Project (RDP) databases at an 80% confidence level (47). After respectively rarefying the bacterial and archaeal OTU tables to the same sequencing depth, alpha diversity indices (ACE, Chao1, Shannon, and inverse Simpson) were calculated using the “vegan” package (v2.5-6) in R (v3.6.3). Chao1 and ACE indices are used to explain the community richness, and Shannon and inverse Simpson indices represent the community diversity.

### Effects of acetate and lactate on the pit mud microbial community.

Pit mud is immersed in the leaching solution of fermented grains (termed *Huangshui* in Chinese) during the baijiu fermentation process. Therefore, we speculate that under the impacts of continuous batch-to-batch brewing activities for decades, perhaps centuries, the formation of pit mud microbiota could be largely affected by the metabolites of the fermented grain microbiota. According to previous work, the major microbial metabolites in fermented grains were lactate and acetate besides ethanol (11), which might be further metabolized to hexanoate and butyrate by the microbial community inhabiting pit mud.

To test the hypothesis, a semisynthetic liquid medium (pH 6.5) was prepared, including 5.0 g/liter acetate (acetate group) or lactate (lactate group), 5.0 g/liter glucose, 1.0 g/liter tryptone, 0.50 g/liter K_2_HPO_4_·3H_2_O, 0.40 g/liter NH_4_Cl, 0.40 g/liter MgSO_4_·7H_2_O, 0.40 g/liter NaCl, 0.050 g/liter CaCl_2_·2H_2_O, 15 ml/liter *huangshui*, and 1 ml/liter micronutrient solution. The composition of micronutrients was as follows (g/liter): nitrilotriacetic acid (1.5), MnSO_4_·H_2_O (0.50), FeSO_4_·7H_2_O (0.10), CoSO_4_·7H_2_O (0.18), ZnSO_4_·7H_2_O (0.18), CuSO_4_·5H_2_O (0.01), KAl(SO_4_)_2_·12H_2_O (0.02), H_3_BO_3_ (0.01), Na_2_MoO_4_·2H_2_O (0.01), NiCl_2_·6H_2_O (0.03), Na_2_SeO_3_·5H_2_O (0.30 mg/liter), and Na_2_WO_4_·2H_2_O (0.40 mg/liter). Five grams of pit mud and 250 ml semisynthetic medium were added in a 500-ml conical flask, followed by purging nitrogen for ∼30 min to maintain anaerobic conditions. Three parallel replicates were performed for each group. The mixture was incubated for 28 days at 30°C in a thermostat. Samples (5 ml) were taken after mixing thoroughly at 0, 6, 18, and 28 days using the sterile syringe, and centrifuged at 5,000 × *g* for 15 min. The supernatant was collected for the analysis of the four main organic acids (27), and the precipitate was obtained for the analysis of prokaryotic community structure and biomass as described previously.

### The effects of fed-batch fermentation with lactate on the evolution of pit mud microbiota.

We further zoomed in on deciphering the role of lactate in the formation of pit mud microbiota due to the following aspects: (i) lactate is the most abundant organic acid in *huangshui*, above 10 times greater than acetate; and (ii) previous study has obtained a microbiota dominated by *Clostridium* cluster IV from pit mud that preferred utilizing lactate for hexanoate biosynthesis (16). Three parallel continuous stirred tank reactors (5 liters) were prepared, and each was with a working volume of 2.0 liters, i.e., the above-mentioned semisynthetic medium with 5 g/liter lactate and without glucose. Fifty grams of pit mud was added in the reactor and incubated at 30°C for 15 days at 100 rpm under an anaerobic environment. For each reactor, 50 ml fermentation liquid was taken every day while adding 50 ml fresh medium and adjusting pH to 6.5. The contents of lactate, acetate, butyrate, and hexanoate in the reactor were measured by HPLC every 24 h, and once lactate was utilized, it was supplied to 5 g/liter. Bacterial community structure was analyzed on day 15 by 16S rRNA gene amplicon sequencing.

### Statistical analysis.

The statistical significance (*P* value < 0.05) of the difference of physicochemical properties, alpha diversity indices, and biomass among the studied groups was determined by one-way analysis of variance (ANOVA) using SPSS 21.0 software. The following analysis was carried out using R (v3.6.3). Constrained principal coordinate analysis (CPCoA) based on Bray-Curtis and Jaccard distance was conducted to differentiate the features of pit mud microbial volatile metabolites. Principal coordinate analysis (PCoA) and cluster analysis of the bacterial and archaeal community structure were conducted using Bray-Curtis (also Jaccard for PCoA) distance metrics. As the first axis length was <3 in detrended correspondence analysis (DCA), redundancy analyses (RDA) between predominant bacterial and archaeal genera (average relative abundance > 1%) and the four main organic acids were conducted with an adjusted *P* value of <0.05 as the threshold of significance. The Spearman correlation coefficients were calculated to analyze the correlation between predominant bacterial and archaeal genera (average relative abundance > 1%) and major volatile compounds (average relative concentration > 50 mg/kg), and a *P* value of <0.05 was considered to determine the significance. Linear discriminant analysis effect size (LEfSe) using relative abundance was applied to discriminate the significantly representative taxa of prokaryotic communities among the pit mud samples with different ages (LDA score ≥ 2) (48). The results of physicochemical properties were visualized with the GraphPad Prism 8 software, and heatmaps were drawn by the “pheatmap” package in R.

### Data availability.

The raw sequencing data sets have been deposited in the BIG Sub Genome Sequence Archive (GSA) (https://bigd.big.ac.cn/gsub/) with the accession number CRA003418 (Bioproject number PRJCA003752).
