# Supplementary material for: Mining the Factors Driving the Evolution of the Pit Mud Microbiome under the Impact of Long-Term Production of Strong-Flavor Baijiu
Source: Appl Environ Microbiol. 2021 Aug 11;87(17):e00885-21. doi: 10.1128/AEM.00885-21 (PMC8357292; doi:10.1128/AEM.00885-21)
Supplement: Supplemental file 1 — Tables S1 to S3, Fig. S1 to S9. Download AEM.00885-21-s0001.pdf, PDF file, 1.5 MB [file aem.00885-21-s0001.pdf]

Mining the evolutionary patterns of pit mud microbiome and driving factors under the impact of long-term strong-flavor *baijiu* production

Li-Juan Chai[b], Wei Qian[a], Xiao-Zhong Zhong[b], Xiao-Juan Zhang[b,d], Zhen-Ming Lu[b,d], Su-Yi Zhang[e], Song-Tao Wang[e], Cai-Hong Shen[e], Jin-Song Shi[c], Zheng-Hong Xu[a,b,e,*]

[a] Key Laboratory of Industrial Biotechnology of Ministry of Education, School of Biotechnology, Jiangnan University, Wuxi 214122, P.R. China

[b] National Engineering Laboratory for Cereal Fermentation Technology, Jiangnan University, Wuxi 214122, P.R. China

[c] School of Pharmaceutical Science, Jiangnan University, Wuxi 214122, P.R. China

[d] Jiangsu Engineering Research Center for Bioactive Products Processing Technology, Jiangnan University, Wuxi 214122, P.R. China

[e] National Engineering Research Center of Solid-State Brewing, Luzhou 646000, P.R. China

*Corresponding author at: National Engineering Laboratory for Cereal Fermentation Technology, Jiangnan University, 1800 Lihu Avenue, Wuxi 214122, P.R. China

E-mail address: zhenghxu@jiangnan.edu.cn (Z.-H. Xu)

Table S1. Summary of bacterial and archaeal 16S rRNA gene sequencing results of different-aged pit mud samples

Sample names		Reads length (bp)	Observed species	Raw reads	Clean reads	Read utilization ratio (%)
**Bacteria**						
Pit wall mud	30_1	295-299	437	150,260	140,076	93.22
	30_2	294-299	463	114,006	106,734	93.62
	30_3	293-299	464	139,586	129,248	92.59
	30_4	299-300	456	141,530	132,642	93.72
	100_1	294-298	530	176,020	159,330	90.52
	100_2	298-300	461	175,052	158,526	90.56
	100_3	298-299	510	130,268	119,178	91.49
	100_4	296-298	545	139,114	126,244	90.75
	100_5	298-299	567	151,114	137,944	91.28
	300_1	297-299	571	158,750	144,452	90.99
	300_2	296-297	461	161,918	146,688	90.59
	300_3	297-299	566	173,326	156,780	90.45
	300_4	297-298	581	146,136	133,594	91.42
Pit bottom mud	30_1	299-299	528	124,862	115,736	92.69
	30_2	296-299	502	146,618	135,448	92.38
	30_3	299-299	582	170,482	155,596	91.27
	30_4	295-298	554	147,776	132,954	89.97
	100_1	298-298	630	168,888	155,214	91.90
	100_2	295-297	673	157,302	145,232	92.33
	100_3	294-297	597	161,252	149,556	92.75
	100_4	293-297	521	146,990	132,896	90.41
	100_5	297-300	431	173,926	158,916	91.37
	300_1	296-299	427	152,534	137,774	90.32
	300_2	296-296	391	141,124	127,882	90.62
	300_3	296-299	418	156,856	142,200	90.66
	300_4	296-298	458	173,902	157,850	90.77
Summary			517.6^{AVG}	$3,979,592^{T}$	$3,638,690^{T}$	91.49^{AVG}
**Archaea**						
Pit wall mud	30_1	298-299	15	160,756	152,826	95.07
	30_2	298-298	19	161,134	153,346	95.17
	30_3	297-298	17	161,374	153,016	94.82
	30_4	296-298	15	164,350	155,178	94.42
	100_1	297-299	15	161,702	152,884	94.55
	100_2	297-297	15	174,058	156,192	89.74
	100_3	296-297	26	167,748	155,852	92.91

	100_4	295-297	24	170,292	154,812	90.91
	100_5	294-297	23	174,780	156,100	89.31
	300_1	296-296	23	169,558	157,178	92.70
	300_2	295-296	21	161,732	153,596	94.97
	300_3	294-296	23	161,460	152,616	94.52
	300_4	293-296	25	184,298	157,524	85.47
Pit bottom mud	30_1	295-298	23	162,220	153,954	94.90
	30_2	294-298	18	171,436	154,732	90.26
	30_3	293-298	22	165,258	155,750	94.25
	30_4	298-300	22	161,562	152,762	94.55
	100_1	293-297	19	173,092	155,372	89.76
	100_2	297-300	21	167,700	153,036	91.26
	100_3	296-299	20	160,016	151,708	94.81
	100_4	296-298	20	158,946	152,532	95.96
	100_5	296-297	18	172,326	155,154	90.04
	300_1	295-296	20	159,122	153,718	96.60
	300_2	295-295	20	159,336	152,366	95.63
	300_3	294-295	16	172,906	156,144	90.31
	300_4	293-295	16	171,110	157,442	92.01
Summary			20.7 ^{AVG}	4,328,272 ^{T}	4,015,790 ^{T}	92.88 ^{AVG}

Note: Read utilization ratio (%) = (Clean reads/Raw reads) × 100%; ^{T}, total reads; ^{AVG}, average value. Numbers 30, 100 and 300 represent the age of fermentation cellar, and numbers 1-5 after the underscores refer to the biological replicates of each sample.

Table S2. Summary of bacterial 16S rRNA gene sequencing results of acetate group, lactate group, and day 15 samples following continuous fermentation with lactate

Sample names	Qualified clean data	Observed species	Chao1	Shannon	Inverse Simpson
Day0_a	35692	264	374.70	3.57	0.82
Day0_b	34536	198	403.06	2.46	0.60
Day0_c	33544	321	396.31	3.33	0.71
**AVG**		**261**	**391.36**	**3.12**	**0.71**
**Acetate group**					
Day6_a	68211	361	468.06	4.87	0.92
Day6_b	67444	355	483.46	4.76	0.89
Day6_c	65411	340	464.03	5.10	0.92
**AVG**		**352**	**471.85**	**4.91**	**0.91**
Day18_a	77733	428	511.72	5.70	0.96
Day18_b	77576	482	627.00	5.80	0.95
Day18_c	77217	466	546.94	6.12	0.97
**AVG**		**458.67**	**561.89**	**5.87**	**0.96**
Day28_a	73503	355	483.27	5.44	0.96
Day28_b	72994	469	625.02	5.79	0.96
Day28_c	71496	480	618.49	5.98	0.97
**AVG**		**434.67**	**575.59**	**5.74**	**0.96**
**Lactate group**					
Day6_a	60948	355	434.02	4.37	0.81
Day6_b	59216	312	399.39	4.29	0.82
Day6_c	56346	395	474.33	5.37	0.93
**AVG**		**354**	**435.92**	**4.68**	**0.85**
Day18_a	64392	424	574.67	4.92	0.88
Day18_b	64152	411	543.25	5.00	0.89
Day18_c	62928	464	622.87	5.68	0.95
**AVG**		**433**	**580.27**	**5.20**	**0.91**
Day28_a	62761	392	530.75	4.60	0.86
Day28_b	62378	463	563.26	5.78	0.95
Day28_c	61792	464	556.50	5.65	0.95
**AVG**		**439.67**	**550.17**	**5.34**	**0.92**
**Continuous fermentation with lactate**					
Day15_a	43826	326	408.83	5.01	0.92
Day15_b	35750	227	306.69	4.74	0.91
Day15_c	36296	290	331.76	4.59	0.90
**AVG**		**281**	**349.09**	**4.78**	**0.91**

AVG, average value; a, b and c are the biological replicates for each sample.

Table S3. Spearman correlation analysis (| r | > 0.6, $P < 0.05$) between main organic acids and bacterial groups in pit mud following the anaerobic fermentation with acetate and lactate

Taxonomy	Lactate	Acetate	Butyrate	Hexanoate
Clostridium	-	-	0.82	-
Caproiciproducens	-	-	-	0.61
Oxobacter	-0.70	-	-	0.87
Uncultured Clostridiaceae Family XI	-0.66	-	-	0.84
Ruminiclostridium	-0.75	-	-	0.92
Uncultured Ruminococcaceae	-0.62	-	-	0.82
Lactobacillus	-	0.72	-	-
Carnobacterium	-	-	-0.63	-0.72
Uncultured Planococcaceae	-	-	-0.75	-
Alcaligenes	0.60	-	-	-
Brevundimonas	-	-	-0.69	-
Corynebacterium	-	-	-0.73	-0.74
Empedobacter	0.80	-	-	-0.83
Other Bacteroidia	-	-	-0.66	-

Fig. S1

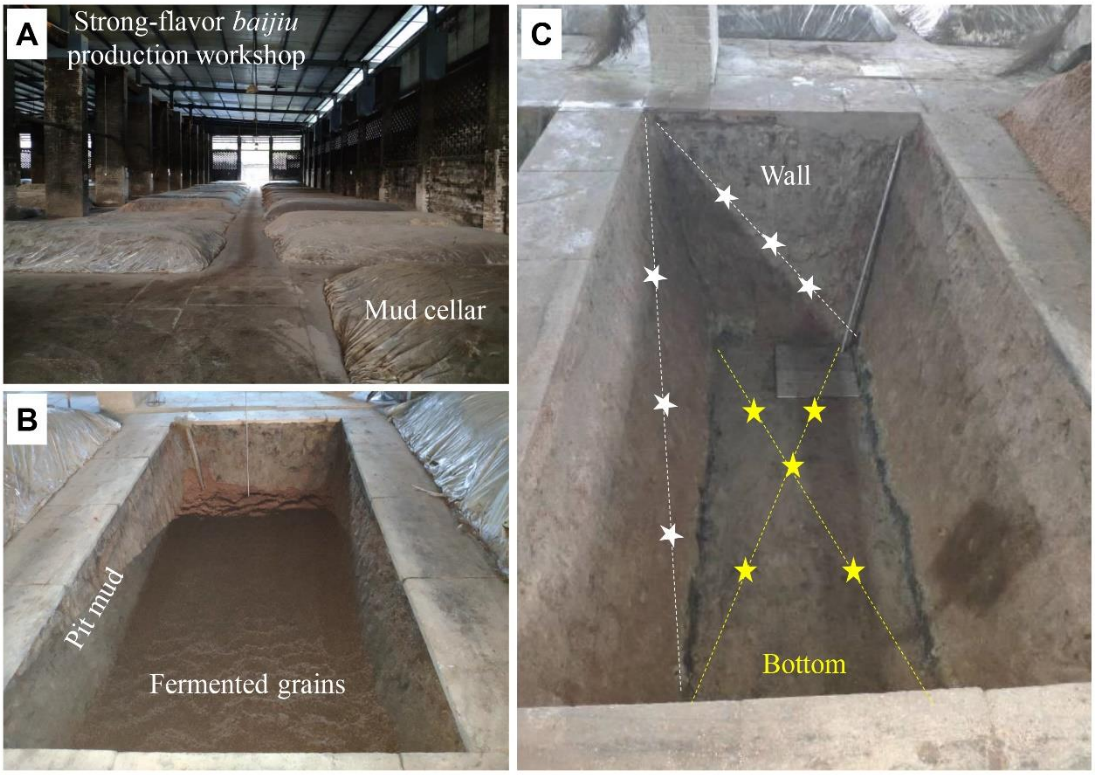

Fig. S1. Photos of strong-flavor *baijiu* (A) production workshop and (B and C) fermentation cellar provided by Luzhou Laojiao Co., Ltd. (Sichuan, China). Mud cellars are covered with polyethylene cloth to keep moist during fermentation. Stars represent the sampling sites.

Fig. S2

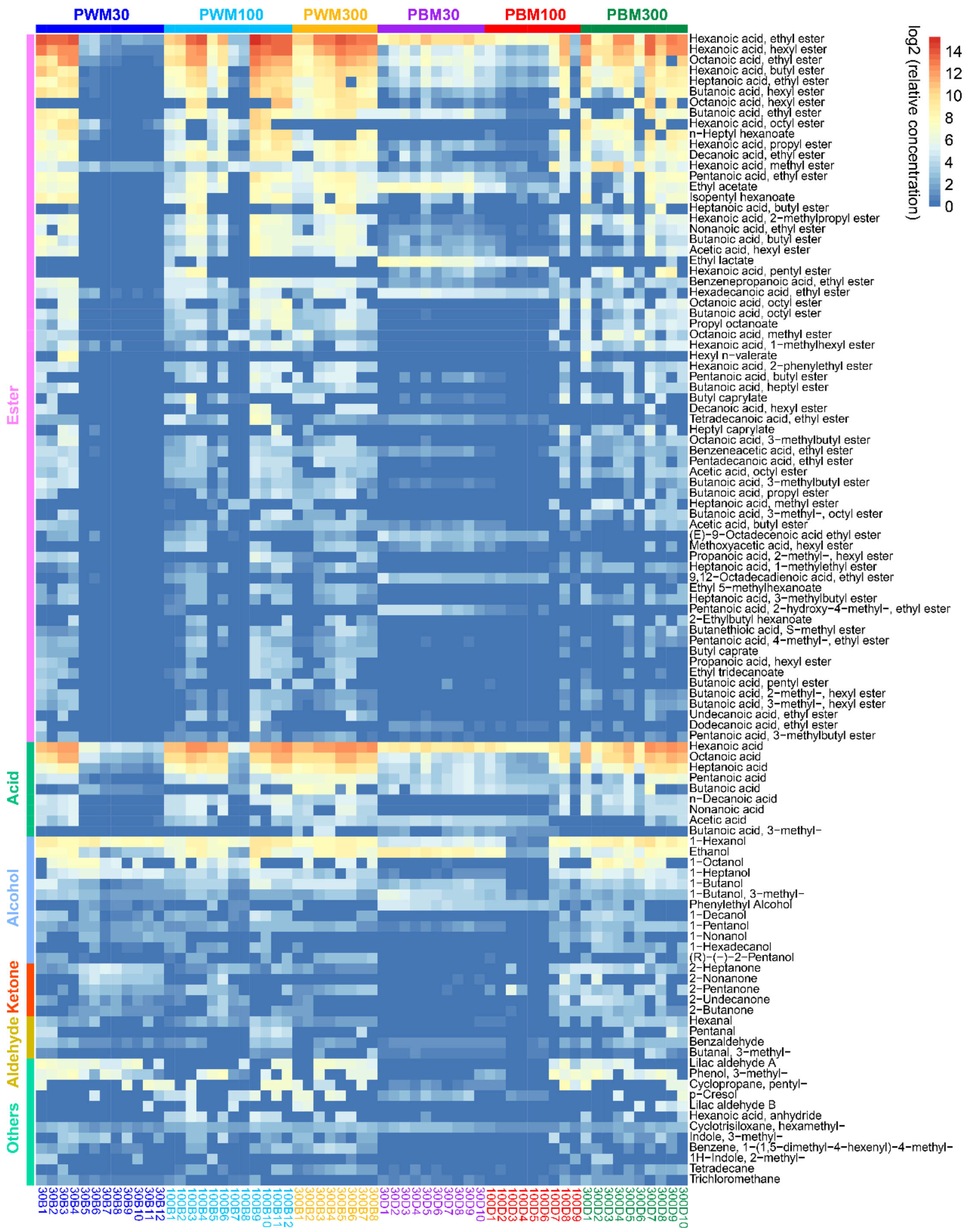

Fig. S2. Microbial volatile compounds identified in the studied pit mud samples by HS-SPME/GC-MS analysis. Values were indicated by $\log_2$ (relative concentration). The letters B and D refer to pit wall mud and pit bottom mud, respectively.

Fig. S3

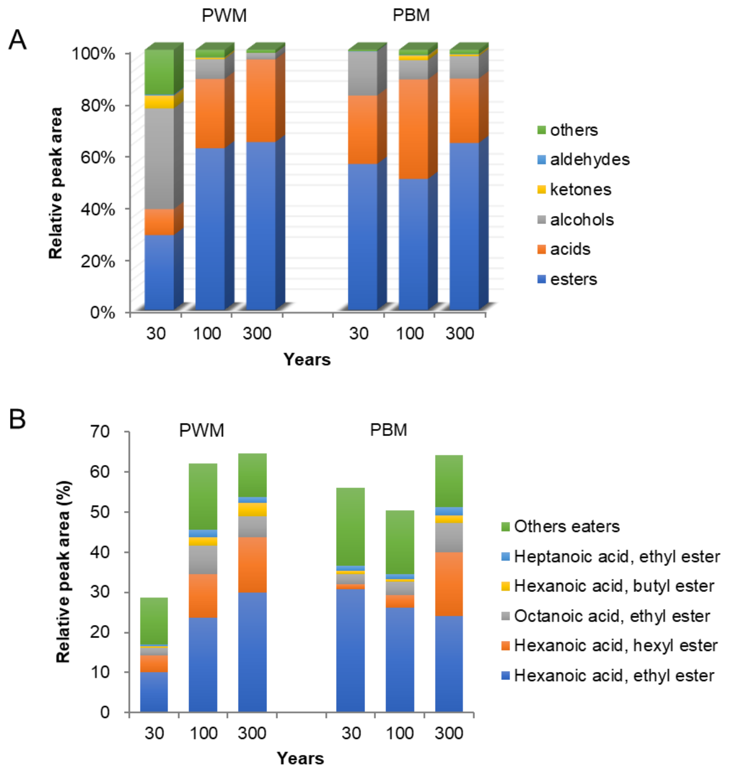

Fig. S3. (A) The distribution across six chemical classes of microbial volatile compounds identified in pit mud samples; (B) The relative peak area of top 5 esters.

Fig. S4

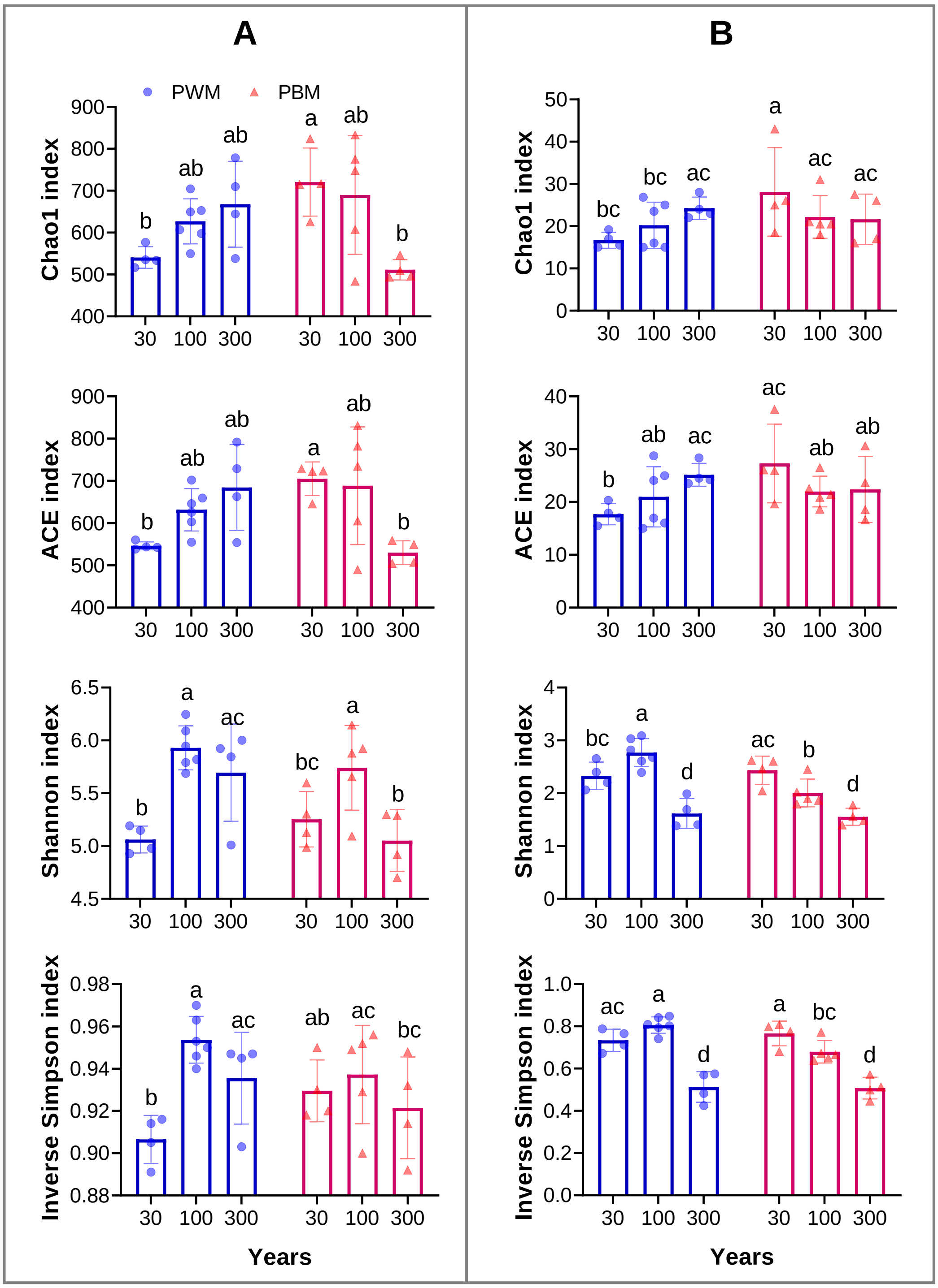

Fig. S4. Alpha diversity analysis of prokaryotic communities in pit mud. (A) Bacterial community; (B) Archaeal community. The significant differences were analyzed by one-way ANOVA ($P < 0.05$).

Fig. S5

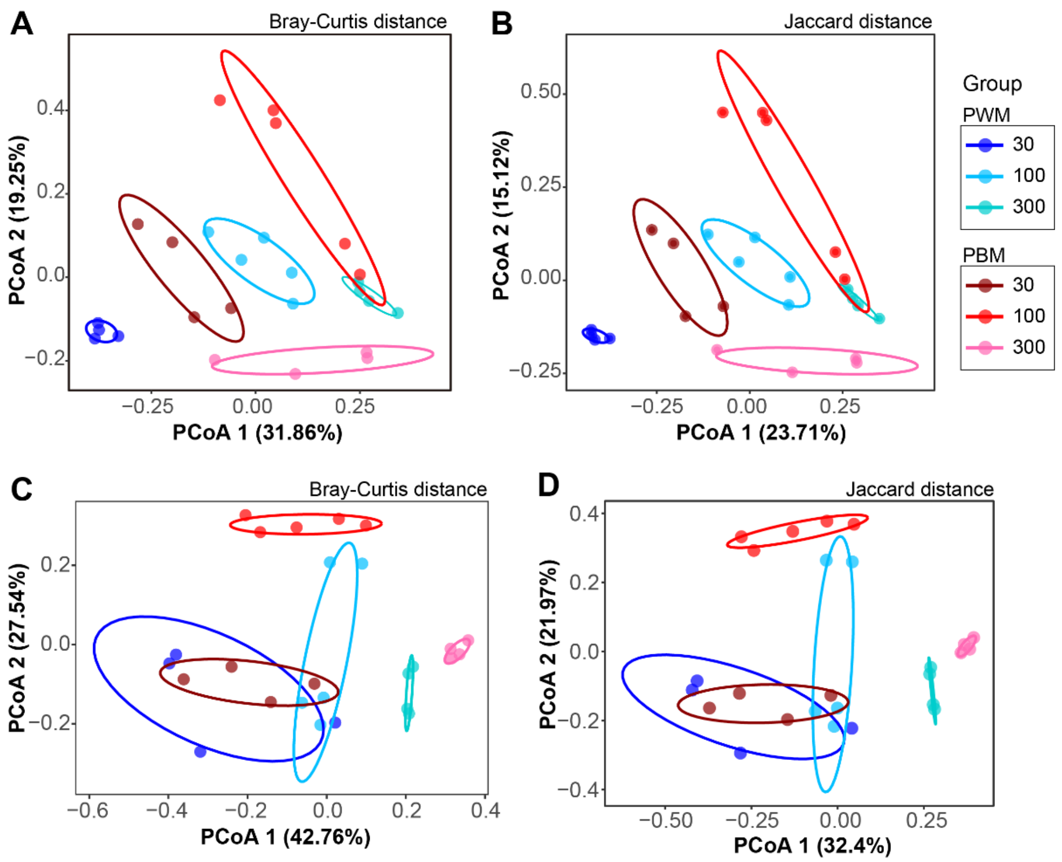

Fig. S5. Principal coordinate analysis (PCoA) of pit mud prokaryotic communities based on Bray-Curtis and Jaccard algorithms. (A) and (B) Bacterial community; (C) and (D) Archaeal community. Numbers 30, 100 and 300 represent the age of fermentation cellar.

Fig. S6

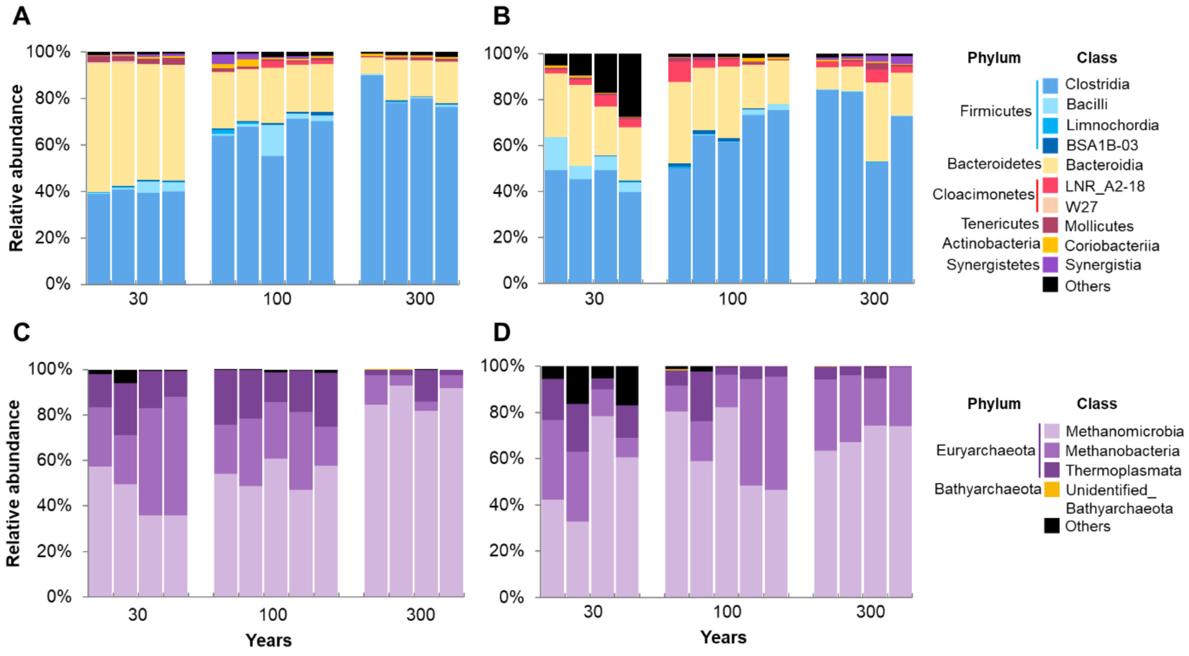

Fig. S6. Taxonomic composition of bacteria and archaea in pit mud at the class level. The relative abundances of bacterial/archaeal classes in (A/C) pit wall mud and (B/D) pit bottom mud samples.

Fig. S7

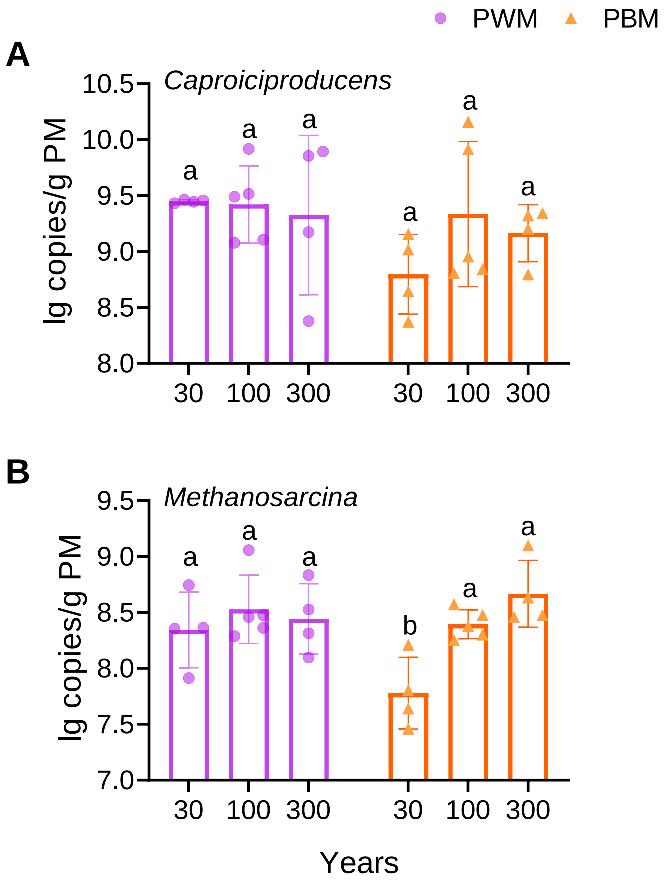

Fig. S7. The absolute abundances of *Caproiciproducens* and *Methanosarcina* in pit mud quantified by the multiplication of relative abundance and total bacterial or archaeal 16S rRNA gene copies.

Fig. S8

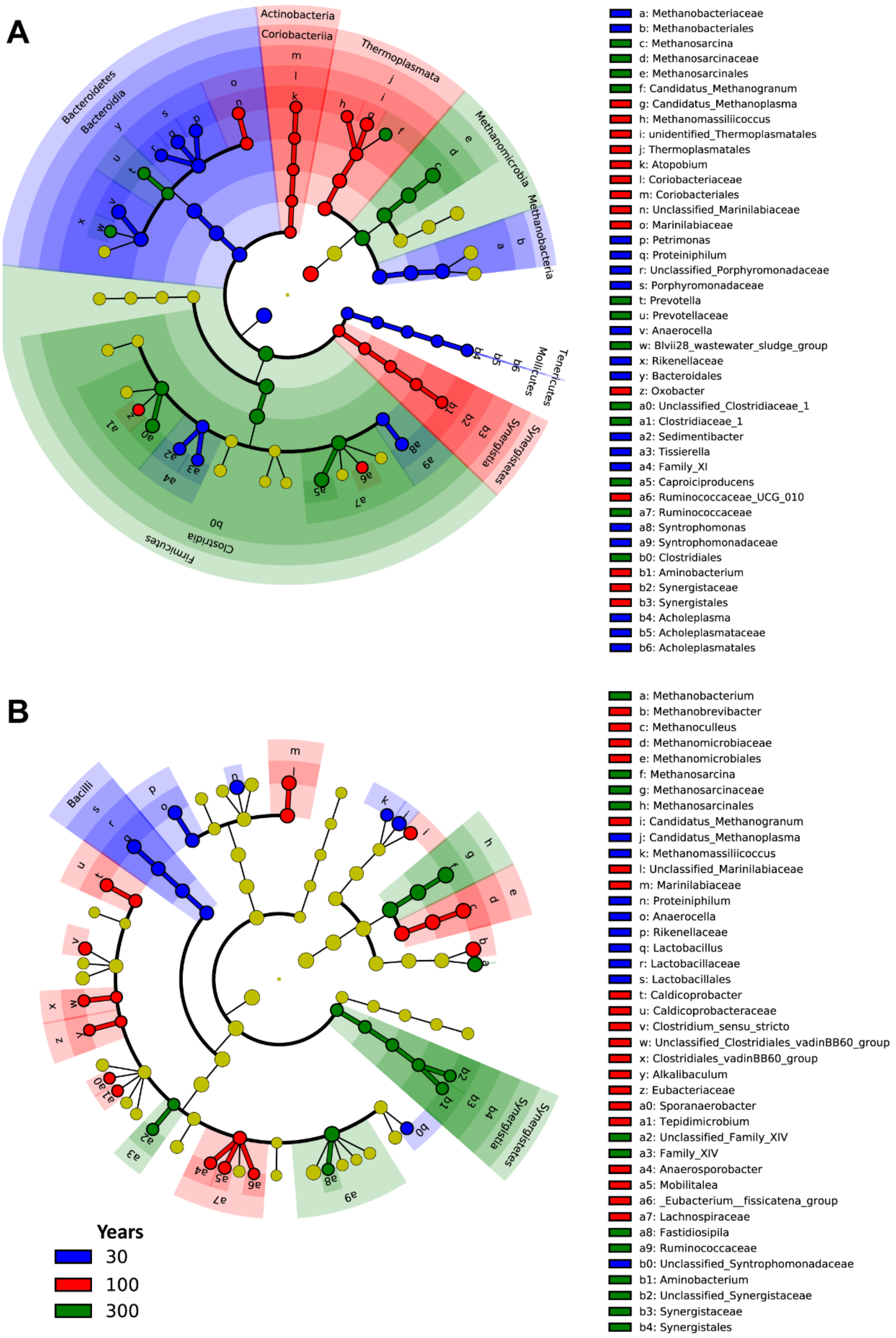

Fig. S8. Linear discriminant analysis effect size (LEfSe) of prokaryotic communities amongst the different-aged pit mud samples (LDA score ≥ 2). (A) Pit wall mud samples; (B) Pit bottom mud samples.

Fig. S9

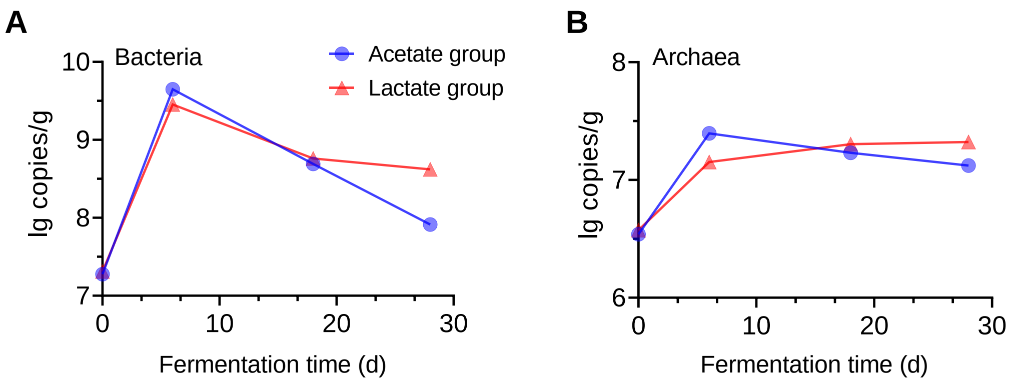

Fig. S9. (A) Bacterial and (B) archaeal biomass in pit mud following the anaerobic fermentation with acetate and lactate by quantitative PCR analysis.
